# The Protective Effect of Low-Dose Ethanol on Myocardial Fibrosis through Downregulating the JNK Signaling Pathway in Diabetic Rats

**DOI:** 10.1155/2016/3834283

**Published:** 2016-07-31

**Authors:** Ying Yu, Xian-Jie Jia, Wei-ping Zhang, Ting-ting Fang, Jie Hu, Shan-Feng Ma, Qin Gao

**Affiliations:** ^1^Department of Physiology, Bengbu Medical College, 2600 Dong Hai Avenue, Bengbu 233030, China; ^2^Department of Epidemiology and Statistics, Bengbu Medical College, 2600 Dong Hai Avenue, Bengbu 233030, China

## Abstract

*Objective*. To investigate the effects of low dose ethanol feeding in diabetic rats and analyze its underlying mechanisms.* Methods*. Male Sprague-Dawley rats were divided into 4 groups: control (Con), diabetes at 4 weeks (DM4W), diabetes at 8 weeks (DM8W), and EtOH + DM8W. After 8 weeks, hemodynamic parameters were recorded and heart weight/body weight (H/B) and hydroxyproline (Hp) content in myocardium were measured. Morphology of collagen in myocardial tissue was observed with Masson's trichrome staining method and collagen volume fraction (CVF) was analysed. The mRNA expression of ALDH2 was assessed with Real-Time PCR. The protein expressions of p-JNK and JNK were evaluated using western blot.* Results*. In contrast to Con group, there was no difference in hemodynamic parameters in DM4W group, but mean arterial pressure and heart rate were decreased in DM8W group, and the ratios of H/B, Hp, and CVF were markedly increased. ALDH2 mRNA expression was decreased, while the ratio of p-JNK/JNK were increased. Compared with DM8W group, the above indexes were improved in EtOH + DM8W group.* Conclusion*. With low dose ethanol intervention, enhanced ALDH2 expression can antagonize the happening of myocardial fibrosis in diabetic rats, which may be relevant with downregulating the JNK pathway.

## 1. Introduction

Diabetes mellitus (DM) is one of the major public health problems around the world [1]. According to the compiled data of the World Health Organization (WHO), approximately 150 million people have diabetes mellitus worldwide, and this number of diabetic patients may be doubled by the year 2025 [2].

Alongside established lifestyle factors, such as smoking, adiposity, and diet, ethanol consumption is thought to play a role in the development of diabetes. Ethanol is a very commonly used chemical substance and also has dose-related effect on cardiac events. Possible cardioprotective effects of ethanol consumption continue to be hotly debated in the medical literatures and popular media [3]. Systematic reviews and meta-analyses have addressed that there is a curvilinear relationship between ethanol consumption and cardiovascular disease, with a protective effect of moderate ethanol consumption and a detrimental effect of large amounts intake [3–7]. Importantly, epidemiological studies suggest that light to moderate alcohol consumption decreases the risk of cardiovascular events, that is, 1-2 drinks per d or 3–9 drinks per wk (one beer, one glass of wine, or one glass of spirit was approximated to one standard drink defined as 1.5 cL or 12 g of pure ethanol [7]). Moderate drinking can activate metabolism of ethanol by acetaldehyde dehydrogenase-2 generation [3] to reduce the incidence of diabetic cardiomyopathy [8, 9].

Diabetic cardiomyopathy is defined as the ventricular dysfunction that occurs in diabetic patients independent of another cause, such as coronary artery disease or hypertension. Diabetic cardiomyopathy is a common complication of diabetes which has become a major cause of diabetes-related morbidity and mortality [10]. Mitochondrial acetaldehyde dehydrogenase-2 (ALDH2) is a key enzyme which plays an important role in the metabolism of acetaldehyde and other toxic aldehydes [11]. Preclinical studies in rats also suggest that ALDH2 activity may affect diabetic pathology [12]. Furthermore, in transgenic mice, overexpression of ALDH2 has been shown to be protective against streptozotocin-induced diabetic cardiomyopathy [13]. Our previous results had indicated that ALDH2 expression was further decreased accompanying the development of diabetes. Meanwhile, we investigated the effects of low-dose ethanol feeding in diabetic rats which could promote myocardial protection through activation of ALDH2 expression. And more, we also found that upregulation of ALDH2 played a protective effect in myocardial ischemia and reperfusion injury and diabetes cardiomyopathy. ALDH2 may be an endogenous cardiac protective factor in myocardial injury [2, 8, 14].

Several biological mechanisms have been proposed to explain that myocardial fibrosis is one of the main pathological changes of diabetic cardiomyopathy. Studies have found C-JUN N-terminal kinase (JNK) signaling pathway played a key role in the process of myocardial fibrosis [15–17]. Evidence had shown that activation of the JNK pathway was involved in the progression of diabetes induced myocardial fibrosis and that such a pathway could be a therapeutic target for diabetic heart injury and cardiomyopathy [18, 19]. However, whether the JNK pathway is involved in the cardioprotective effect of low-dose ethanol on diabetic rats has not been fully elucidated.

So in this study, we mimic diabetes model by intraperitoneal injection of streptozotocin (STZ) in combination with low-dose ethanol; the purpose of the present study is as follows: (1) to investigate the mechanism of low-dose ethanol which alleviates myocardial fibrosis in diabetic cardiomyopathy; (2) to clarify whether low-dose ethanol mediated protection is associated with downregulating the JNK signaling pathway. This study might shed some light on low-dose ethanol as an effective therapeutic in the treatment of diabetic cardiomyopathy.

## 2. Methods

### 2.1. Animals

Adult male Sprague-Dawley rats (200 to 250 g) were obtained from Bengbu Medical College Animal Administration Center. All animal studies were approved by the Animal Ethics Committee of Bengbu Medical College and performed in accordance with the ethical standards. The rats were fed normal chow and had free access to distilled water.

### 2.2. Chemicals and Reagents

Streptozotocin (STZ) was purchased from Sigma (USA). TRIzol was purchased from Invitrogen (USA); hydroxyproline (Hp) was purchased from Nanjing Jiancheng Bioengineering Institute (China). Ethanol (EtOH) was purchased from Bengbu New Chemical Reagent Factory (China). *β*-actin antibodies were purchased from Santa Cruz Biotechnology (USA), rabbit c-Jun N-terminal kinase (JNK) and phosphorylated JNK (p-JNK) were purchased from Anbo Biotechnology (USA). Chemiluminescence reaction (ECL) system was purchased from Millipore, Billerica (USA). All primers were purchased from Shanghai Sangon Biotech (China).

### 2.3. Induction of Diabetes and Experimental Protocol

As previously described by our laboratory, STZ at 55 mg/kg freshly dissolved in 0.1 mol/L sodium citrate buffer (pH 4.5) was injected intraperitoneally to induce diabetic models in overnight fasted rats [8]. All rats were randomly divided into four groups: normal control group (Con), diabetes at 4 weeks group (DM4W), diabetes at 8 weeks group (DM8W), and ethanol + diabetes at 8 weeks group (EtOH + DM8W), respectively (*n* = 6). In Con group, rats were fed with standard rat chow and received an intraperitoneal injection of the same volume of citrate buffer for 8 weeks. In EtOH + DM group, DM rats were fed with 2.5% EtOH in their drinking water for one week to initiate drinking then, it was changed to 5% EtOH continuous access through the remaining 7 weeks.

### 2.4. Hemodynamics

Male SD rats were anaesthetized by use of chloral hydrate (100 mg/kg) through intraperitoneal injection. Throughout the experiment, systolic pressure (SP), diastolic pressure (DP), mean arterial pressure (MAP), and heart rate (HR) were determined by invasive hemodynamic evaluation methods for 30 min. At the end of the experimental period, hearts were excised rapidly, placed in ice-cold Krebs-Henseleit (K-H) buffer, and weighed and the ratio of heart weight/body weight (H/B) was calculated.

### 2.5. Detection of Hydroxyproline Content

Heart tissue (100 mg) was homogenized in ice-cold K-H buffer. The supernatant was collected after centrifugation for 20 min (2000 rpm). The protein concentration was measured by the Bradford method. Hydroxyproline (Hp) content was detected according to the instruction manual.

### 2.6. The Content of Collagen Detection by Masson-Staining

Left ventricular tissue obtained from all groups was stained with Masson's trichrome for the quantification of collagen. The histological sections were taken in 10% neutral formalin-fixed, dehydrated, paraffin embedded sections. Then, the samples underwent the dehydration of gradient ethanol, neutral resin embedding, and Masson trichrome staining. The collagen fibers were stained blue and cardiomyocytes were stained red. Myocardial collagen was quantified at a final magnification of 200x with a polarized microscope connected to a video camera. Myocardial collagen volume fraction (CVF) was analyzed using Image Pro analysis software, expressed as the mean percentage of collagen area to the total area of each microscopic field. Five visions under microscope of each sample were randomly chosen and the average of them was taken for analysis [17].

### 2.7. Detection of ALDH2 mRNA by Real-Time PCR

Total RNA was extracted from the left anterior myocardium using TRIzol according to the manufacturer's instructions. Total RNA (3 *μ*g) was reversely transcribed to cDNA, and PCR was performed by a routine method. The primers used were as follows: for ALDH2, forward: 5-GTG TTC GGA GAC GTC AAA GA-3′ and reverse: 5′-GCA GAG CTT GGG ACA GGT AA-3′ and the product size was 187 bp; for *β*-actin, forward: 5′-GAG ACC TTC AAC ACC CCA GCC-3′ and reverse: 5′-GGC CAT CTC TTG CTC GAA GTC-3′ and the product size was 312 bp [8]. The PCR condition was as follows: predenaturing at 95°C for 3 min and then 40 cycles (50 s denaturation at 95°C, 50 s annealing at 62.5°C, and 60 s extension at 72°C), followed by a final step at 72°C for 10 min. Real-Time PCR was performed to determine the ALDH2 mRNA level.

### 2.8. Detection of JNK and p-JNK Protein Expressions by Western Blot

Myocardium tissues (100 mg) from each group were collected and homogenized in a lysis buffer. Homogenates were sonicated and centrifuged at 12,000 ×g for 30 min at 4°C. The protein concentration was determined using the bicinchoninic acid (BCA) Protein Assay kit. Total protein (80 *μ*g) was separated by SDS-polyacrylamide gel electrophoresis (PAGE) and transferred electrophoretically to a polyvinylidene difluoride (PVDF) filter membrane [20].

The membranes were blocked with 5% nonfat milk in Tris-buffered saline Tween (TBST) for 2 h, and then they were incubated at 4°C overnight with the corresponding primary rabbit JNK antibody (1 : 1000), rabbit p-JNK antibody (1 : 1000), and mouse *β*-actin antibody (1 : 500). All membranes were incubated for 1 h with corresponding secondary antibody HRP-linked anti-mouse IgG or HRP-linked anti-rabbit IgG. Autoradiographs were scanned and the band density was determined with Image J software.

### 2.9. Statistical Analysis

Data were expressed as mean ± SD. One-way analysis of variance (ANOVA) followed by Student-Newman-Keuls (SNK) was used for multiple comparisons. *P* < 0.05 was considered as statistically significant.

## 3. Results

### 3.1. Hemodynamics

In contrast to Con group, there was no statistical difference about hemodynamic parameters in DM4W groups, but systolic pressure (SP), diastolic pressure (DP), mean arterial pressure (MAP), and heart rate (HR) were decreased significantly in DM8W groups. Compared with DM8W group, SP, DP, MAP, and HR were rather increased significantly in EtOH + DM8W group. (Table 1)

### 3.2. The Ratio of Heart Weight to Body Weight and Hydroxyproline Content in Myocardial Tissue

Compared with Con group, there was no significant difference about the ratio of heart weight to body weight (H/B) and hydroxyproline (Hp) content in myocardial tissue in diabetic rat in DM4W group; however, with extended duration, H/B ratio and myocardial Hp content were increased in DM8W group, and the difference was statistically significant (Table 2). Compared with DM8W group, H/B ratio and myocardial Hp content were significantly deceased in EtOH + DM8W group (*P* < 0.05).

### 3.3. The Content of Collagen Detection by Masson-Staining

In Masson trichrome staining, the collagen fibers were stained blue and cardiomyocytes were stained red. The collagen tissue was appropriately arranged among cardiomyocytes in control group. However, collagen tissue was increased markedly and disrupted in some area in diabetic group.

In DM4W group, myocardial cells were approximately arranged well, collagen fibers were sparsely distributed, and interstitial collagen was dyed a little blue. Compared with control group, myocardial cells were in a disordered arrangement, the interstitial collagen were edematous, and collagen fibers were unevenly distributed and increased markedly in DM8W group. Compared with DM8W group, myocardial cells were arranged neatly, and collagen fibers were significantly reduced in EtOH + DM8W group.

**Figure 1:**
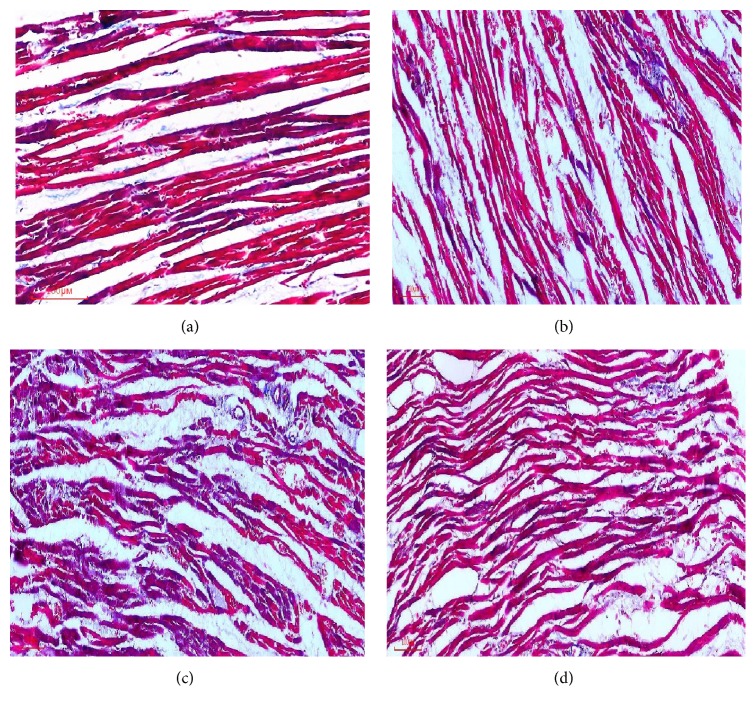
The result of collagen detection by Masson-staining (200). (a) Control, (b) DM4W, (c) DM8W, and (d) EtOH + DM8W.

*Quantitative Analysis Results*. The content of collagen in diabetic group was higher than that of control group. As displayed in Table 2, compared to control group, the contents of collagen volume fraction (CVF) were significantly increased in DM4W group (*P* < 0.05), and with the development of diabetes, CVF were further increased in DM8W groups (*P* < 0.01). But in contrast to DM8W group, the contents of CVF were markedly decreased in EtOH + DM8W group (*P* < 0.01, Figure 1, Table 2).

### 3.4. Changes of Myocardial ALDH2 mRNA Expression

Real-Time PCR revealed that, compared with control group, the expression of ALDH2 mRNA level was reduced in DM4W (*P* < 0.05). With the development of diabetes, ALDH2 mRNA were further decreased in DM8W groups (*P* < 0.01). In contrast to DM8W group, the expression of ALDH2 mRNA was increased in EtOH + DM8W group (*P* < 0.01, Table 3).

### 3.5. Changes of Myocardial JNK and Phospho-JNK Protein Levels

**Figure 2:**
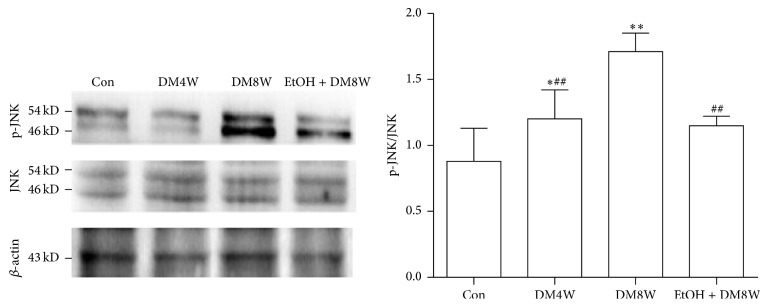
The result of myocardial JNK and phospho-JNK protein: ^*∗*^ *P* < 0.05 and ^*∗∗*^ *P* < 0.01 compared with Con; ^#^ *P* < 0.05 and ^##^ *P* < 0.01 compared with DM8W.

On western blot analysis, compared with control group, the ratio of p-JNK/JNK protein expression was increased in DM4W (*P* < 0.05), and with the development of diabetes, the ratio of p-JNK/JNK was further increased in DM8W groups (*P* < 0.01). Compared with DM8W group, the ratio in EtOH + DM8W group was decreased (*P* < 0.01, Figure 2, Table 3).

## 4. Discussion

Diabetes mellitus is becoming an epidemic health threat and represents one of the most prevalent chronic noncommunicable disorders [21]. Cardiovascular disease is a serious complication of diabetes and is responsible for 80% of the deaths among diabetics [22]. Diabetic cardiomyopathy is one of the most common complications of diabetes; its major pathological characteristics are hypertrophy or hyperplasia of cardiac myocytes. Excessive deposition of myocardial interstitial collagen and myocardial fibrosis often leads to cardiac hypertrophy and decrease of heart function, which play a vital role in the occurrence and development of diabetic cardiomyopathy [23].

In the present study, our results demonstrated that, with the progression of diabetes, systolic pressure, diastolic pressure, mean arterial pressure, and heart rate were declined significantly in DM8W group compared with Con group, but the ratio of heart weight/body weight, hydroxyproline content, and CVF were markedly increased. Meanwhile, the mRNA expression of ALDH2 was decreased and the ratio of p-JNK/JNK was increased. When the DM rats were treated with ethanol at low concentration, hemodynamic parameters were improved and hydroxyproline content and CVF were decreased, accompanied with the increase of myocardial ALDH2 mRNA expression and decrease of p-JNK/JNK protein expression. The results suggested that with low-dose ethanol intervention, enhanced ALDH2 expression can antagonize the happening of myocardial fibrosis in diabetic cardiomyopathy, which may be relevant with downregulating the JNK pathway.

Mitochondrial aldehyde dehydrogenase 2 (ALDH2) is a kind of aldehyde oxidase that is involved in ethanol metabolism, which is closely related to human alcohol drinking behavior [24]. Heavier alcohol consumption is associated with increased risk of developing diabetes mellitus, hypertension, and cardiovascular and cerebrovascular disease [25]. Nevertheless, there are increasing reports showing a critical role for light to moderate alcohol ingestion could protect against myocardial injuries. Researches indicated a U-shaped relationship between alcohol consumption and cardiovascular disease mortality [6, 26]. Statistics manifested the mortality of drinking alcohol up to or over 6 cups (355 mL/cup) per day is 1.6 times as high as control, while light-to-moderate drinking of 1~2 cups alcohol per day [fewer than 2 cups or 1 ounce (28.3495 g)] reduced mortality from cardiovascular disease [27]. In addition, clinical data showed that moderate drinking can protect metabolic syndrome (cardiovascular disease and diabetes) via influencing various factors to reduce the diabetes prevalence, such as glycosylated hemoglobin, high density lipoprotein cholesterol, and fibrinogen [28]. After continuously feeding C57BL/6 mice with 18% of ethanol for 12 weeks, the myocardial mechanics were restored and the expression and activation of PKC and Akt were increased, which established that EtOH feeding causes cardiac expression of activated PKC-*ε* [29]. Sun et al. observed that ischemic injury could result in downregulation of mitochondrial ALDH2 in mice hearts inducing an elevation of aldehyde 4-hydroxy-2-nonenal (4-HNE), leading to cardiomyocyte apoptosis through downregulation of HSP70 and activation of JNK and p53. ALDH2 detoxifies 4-HNE, a mediator of programmed cell death events, by transmitting a mitochondrial ALDH2 signal to elicit a cytosolic response through the JNK/p53 pathway. However, using transgene technology to increase the expression of ALDH2 can contribute to antagonizing heart failure and decreasing heart function [30]. Furthermore, our previous research found that there was a close relationship between ALDH2 and oxidative damage. With the progression of diabetes, the myocardial antioxidant ability of diabetic rats was decreased, which exacerbated the progression of myocardial fibrosis [8, 13, 31]. In this study, we found that the ratio of heart weight/body weight, hydroxyproline content, and CVF were markedly increased in diabetic 4 and 8 weeks' rats, which suggested diabetic induced myocardial fibrosis. When the diabetic rat was treated with low doses of ethanol to induce ALDH2 activity, the hemodynamic parameters were increased and hydroxyproline content and CVF were decreased, which indicated that increasing ALDH2 expression can attenuate the happening of myocardial fibrosis and the destroying of myocardial injuries.

Mitogen-activated protein kinases (MAPKs) play an important role in the signal transduction pathways from the membrane to intracellular compartments including the nucleus. They regulate the functions of many gene products and therefore affect cell growth, differentiation, and apoptosis [32]. Oxidative stress, inflammation, endoplasmic reticulum stress, and autophagy defect can activate MAPKs signaling pathway in the progression of diabetes complications. Li et al. observed that production of a large number of ROS was thought to be an important contributing factor, concomitant with activation of JNK, p38 MAPK, and TGF-*β* in the development and the progression of diabetic cardiomyopathy [16].

C-Jun NH2-terminal kinase (JNK) is one of the major members of MAPKs, and JNK activation is also implicated in cardiac fibrosis [33, 34]. JNK signaling pathway plays a key role in the growth of cardiac fibroblasts induced by high glucose. Studies of diabetic rats had demonstrated that myocardial fibrosis was developed; meanwhile, JNK mRNA expression level and activity were upregulated [17]. Furthermore, disruption of the JNK protein kinase decreased the occurrence and development of diabetic myocardial fibrosis [18, 35]. It is worthwhile to note that, in our experiment, we found that, with the progression of diabetes, the expression of myocardial ALDH2 at mRNA level was decreased and the ratio of p-JNK/JNK at protein level was increased, which suggested ALDH2 and JNK signaling pathway both participated in the occurrence and development of diabetic cardiomyopathy. Moreover, further enhancing activation of ALDH2 expression of low doses of ethanol for 8 weeks in diabetic rats, accompanied with the high-expression of ALDH2, p-JNK/JNK were decreased in contrast to diabetes rats, which suggested activation of ALDH2 expression might be associated with downregulating the JNK signaling pathway to relieve myocardial fibrosis and myocardial injuries.

However, there were some experimental limitations in our study. We only observed that the protection of low-dose ethanol was relevant with downregulating the JNK pathway in diabetic cardiomyopathy. To better understand the mechanisms involved, we will adopt the activation or inhibition of JNK to investigate the downstream signaling molecules in the follow-up experiments. To address this issue, we will use JNK inhibitor to observe whether inhibiting JNK pathway can play the cardiovascular role in diabetes rats. Moreover, we further explore whether enhanced activation of JNK signaling pathway can antagonize the cardioprotective effect of activation of ALDH2 by low-dose ethanol. Through these experiments, we want to verify low-dose ethanol could attenuate myocardial fibrosis via downregulating the JNK pathway in diabetic cardiomyopathy.

In summary, our results demonstrate that with the progression of diabetes, ALDH2 expression was decreased accompanied with the happening of myocardium fibrosis. Treatment with ethanol at low concentration can protect the heart by upregulating ALDH2 and downregulating the JNK signaling pathway against myocardial fibrosis in diabetic cardiomyopathy.

## Figures and Tables

**Table 1:** Hemodynamic data in rats.

Group	SP (mmHg)	DP (mmHg)	MAP (mmHg)	HR (beats/min)
Con	111.09 ± 6.05	95.47 ± 7.75	100.68 ± 7.15	462.63 ± 65.20
DM4W	105.30 ± 4.74^##^	92.76 ± 4.90^##^	96.94 ± 4.85^##^	433.00 ± 14.39^#^
DM8W	83.24 ± 9.32^*∗∗*^	62.97 ± 10.56^*∗∗*^	69.72 ± 9.40^*∗∗*^	377.06 ± 39.04^*∗∗*^
EtOH + DM8W	103.74 ± 4.38^##^	80.45 ± 5.90^##^	88.21 ± 5.37^##^	411.44 ± 22.27^*∗*^

Values are means ± SD (*n* = 6).

Con: normal control group, DM4W: diabetes at 4 weeks group, DM8W: diabetes at 8 weeks group, and EtOH + DM8W: ethanol + diabetes at 8 weeks group.

^*∗*^ *P* < 0.05, ^*∗∗*^ *P* < 0.01 compared with Con; ^#^ *P* < 0.05, ^##^ *P* < 0.01 compared with DM8W.

**Table 2:** Changes of heart weight/body weight (H/B), hydroxyproline (Hp) content, and collagen volume fraction (CVF) in different groups.

Group	H/B (mg/g)	Hp (umol/mg)	CVF (%)
Con	3.76 ± 0.15	0.27 ± 0.05	12.50 ± 0.98
DM4W	3.88 ± 0.20^##^	0.35 ± 0.07	15.74 ± 1.53^*∗*##^
DM8W	4.78 ± 0.10^*∗∗*^	0.43 ± 0.04^*∗∗*^	25.75 ± 1.98^*∗∗*^
EtOH + DM8W	3.96 ± 0.17^##^	0.34 ± 0.05^#^	14.43 ± 2.65^##^

Values are means ± SD (*n* = 6).

^*∗*^ *P* < 0.05 and ^*∗∗*^ *P* < 0.01 compared with Con; ^#^ *P* < 0.05 and ^##^ *P* < 0.01 compared with DM8W.

**Table 3:** The result of ALDH2 mRNA and p-JNK/JNK protein expression.

Group	ALDH2 mRNA	p-JNK/JNK
Con	0.85 ± 0.16	0.88 ± 0.25
DM4W	0.56 ± 0.17^*∗*##^	1.20 ± 0.22^*∗*##^
DM8W	0.21 ± 0.0^*∗∗*^	1.71 ± 0.14^*∗∗*^
EtOH + DM8W	0.61 ± 0.18^##^	1.15 ± 0.07^#^

Values are means ± SD (*n* = 6).

^*∗*^ *P* < 0.05 and ^*∗∗*^ *P* < 0.01 compared with Con; ^#^ *P* < 0.05 and ^##^ *P* < 0.01 compared with DM8W.
